# A new nanosensor composed of laminated samarium borate and immobilized laccase for phenol determination

**DOI:** 10.1186/1556-276X-9-76

**Published:** 2014-02-15

**Authors:** Ping Hu, Xinlin Zhou, Qingsheng Wu

**Affiliations:** 1 Department of Chemistry; Key Laboratory of Yangtze River Water Environment, Ministry of Education, Tongji University, 1239 Siping Road, Shanghai 200092, People’s Republic of China

**Keywords:** SmBO_3_, Nanosheets, Laccase, Immobilization, Determination

## Abstract

A new nanosensor composed of laminated samarium borate and immobilized laccase was developed for phenol determination. The laminated samarium borate was synthesized by a mild solid-state-hydrothermal (S-S-H) method without any surfactant or Template. X-ray diffraction (XRD), Fourier transform infrared spectroscopy (FTIR), and scanning electron microscopy (SEM) were used to characterize the samples. The morphology of the as-prepared materials was characterized by SEM, which shows that laminated samarium borate are uniform nanosheets with a layer-by-layer self-assembled single-crystal structure. These laminated samarium borate have typical diameters of 3 ~ 5 μm and the thickness of each layer is in the range of 10 ~ 80 nm. And then, these SmBO_3_ multilayers were used to immobilize the laccase. The proposed nanosensor composed of laminated samarium borate and immobilized laccase was successfully developed for phenol determination. Cyclic voltammetry were used to study the nanosensor. The proposed nanosensor displayed high sensitivity toward phenolic compounds. The linearity of the nanosensor for the detection of hydroquinone was obtained from 1 to 50 μM with a detection limit of 3 × 10^-7^ M (based on the S/N = 3).

## Background

With the development of the economy, more and more pollutants are eroding the human survival environment. Then the detection and treatment of environmental pollutions have aroused great attentions of scientists. Belonging to multicopper proteins, laccases are widely existed in nature especially fungi [1,2]. It is a phenol oxidase that can catalyze oxidation of many organic pollutants in water [3]. Wan and his group [4] had elaborated the progress on the research of laccases, namely the active center of copper ions, the three-dimensional structure of protein, and its catalytic mechanism. Substrate specificity of laccases was exploited to remove pollutants from the environment without creating the negative effects associated with many other methods [5,6]. It is well known that the enzyme is often easily inactivated in practical applications due to complex environment conditions, which limit its further industrial application [7,8]. Consequently, immobilized laccases have received much attention from researchers in recent years because of its substantial advantages over free laccases such as continuous reuse, easy separation of the product from the reaction media, easy recovery of the enzyme, and improvement in enzyme stability. Nowadays, many different types of methods have been employed in the immobilization of enzymes, such as adsorption, entrapment, cross-link, and covalent attachment. Recently, it is reported that laccase has been successfully immobilized [9-11] on many different types of supports, such as activated carbon [3], magnetic chitosan [12], alginate chitosan [13], porous glass [14], chitosan/poly(vinyl alcohol) composite nanofibrous membranes [15], cellulose-polyamine composite [16], alginate, kaolinite, polymer beads and membranes polystyrene microspheres, short-range ordered aluminum hydroxide, and so on [17-20]. However, leakage, desorption, and the loss of enzyme activity were major problems in laccase immobilization, which was related to many factors involving the enzyme itself, polymer matrix, reaction reagents, and process conditions [9]. Therefore, it is of great interest in developing novel technologies on laccase immobilization to improve catalytic activity of laccase and increase its industrial application.

Among those laccase supports, inorganic materials are more attractive because of their regular structure, good mechanical, chemical, and thermal stabilities [21-23]. Nanomaterials have attracted increasing attention for their novel properties and potential applications with small dimensions [24,25]. Inorganic nanomaterials of rare-earth borate compounds show high vacuum ultraviolet (VUV) transparency and exceptional optical damage thresholds. Acentric lanthanide borate crystals are useful in a wide variety of photonic devices for unique optical, nonlinear optical, laser, electronic, and other physical properties [24,25]. In the past decades, the rare-earth borates are widely used in many fields [26-30] and a number of synthetic methods have been employed to fabricate them. However, many routes suffer from the use of high temperature, tedious processes, and environmental pollution. Therefore, it is still an attractive and necessary topic for the development of environmentally friendly, facile, and reproducible methods to fabricate rare-earth borate nanometer materials.

In this paper, we choose a novel laminated SmBO_3_ multilayer as support for the immobilization of laccase. The SmBO_3_ multilayer samples were synthesized via the solid-state-hydrothermal (S-S-H) method, which exhibits many advantages, such as no side products, facile operation, and low cost. Then laccase was immobilized in SmBO_3_ nanosheets for the fabrication of the nanosensor. The performance of the proposed nanosensor composed of the laminated samarium borate and immobilized laccase in the catalytic determination of phenolic compounds has been investigated in detail.

## Methods

### Reagents and apparatus

All reagents were analytical grade in the synthesis system. H_3_BO_3_ (>99.0%), Sm_2_O_3_ (>99.99%), Na_2_HPO_4_ · 12H_2_O (>99.0%), C_6_H_8_O_7_ · H_2_O (>99.8%), hydroquinone (>99.99%), and 2, 6-dimethoxyphenol (>99.99%) were purchased from Shanghai Chemical Reagent Co, Ltd. (Shanghai, China) and used without any purification. Laccase was provided by Shanghai Daidi Industrial Development Co, Ltd. (Shanghai, China) and stored at 4°C before using.

The morphology and structure of the samples were inspected by using a field emission scanning electron microscope (FE-SEM, Hitachi S4800, Tokyo, Japan) at an accelerating voltage of 5 KV. The phase purity and crystallinity of the samples were characterized by X-ray powder diffraction (XRD) performed on a D8 FOCUS diffractometer (Bruker, Madison, WI, USA) with CuK*α* radiation (*λ* = 0.154056 nm), employing a scanning rate of 0.02° · s^-1^, in the 2*θ* ranges from 10° to 70°. Infrared spectra (4,000 to 400 cm^-1^) are recorded by Nicolet 5DX Fourier transform infrared spectroscopy (FTIR; Thermo Fisher Scientific, Waltham, MA, USA) equipped with a TGS/PE detector and a silicon beam splitter with 1 cm^-1^ resolution.

Electrochemical experiments were carried out with a CHI-660B electrochemical workstation (Shanghai, China). Measurements were performed at least three times on a glassy carbon electrode (GCE). A conventional three-electrode system was employed, comprising a GCE (3-mm diameter) as the working electrode, a platinum wire as the auxiliary electrode, and an Ag/AgCl (saturated KCl) as the reference electrode. Voltammetric responses were recorded in 50 ml of substrate solutions prepared in PBS buffer solution. First, the modified electrode was activated by several successive voltammetric cycles from -0.20 to 0.80 V. Second, cycle voltammograms (CVs) at the rate of 50 mV · s^-1^ were carried out from -0.20 to 0.80 V after subtracting the background. Finally, the GCE was regenerated by 10 successive cyclic voltammetric sweeps in the blank solution. After several measurements, the GCE should be repolished. All the electrochemical measurements were carried out at room temperature.

### Preparation of SmBO_3_ nanocrystals

**Figure 1:**
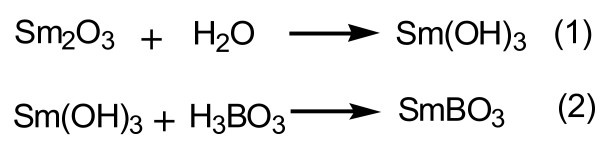
**Formation mechanism of SmBO**_**3**_** in the S-S-H route.**

Precursor-laminated SmBO_3_ multilayers were synthesized by solid-state-hydrothermal method. In a typical synthesis, 0.6 mmol Sm_2_O_3_, 0.72 mmol H_3_BO_3_, 14 ml deionized water are mixed in a 20-ml-capacity Teflon-lined autoclave. The autoclave is sealed and maintained at 200°C constantly for 36 h and then cooled to room temperature naturally. The precipitation is centrifuged and washed with deionized water several times. Finally, as-obtained products are dried under vacuum at 60°C for 4 h. We propose that the formation processes of SmBO_3_ in the solid-state-hydrothermal system at 200°C can be assigned to two stages: Sm_2_O_3_ is first transformed into hydroxide, Sm(OH)_3_, then the hydroxide interacts with H_3_BO_3_ to form products. The formation reactions of SmBO_3_ are proposed and shown in Figure 1.

### Immobilization of laccase on SmBO_3_ nanocrystals

The SmBO_3_ multilayers were employed as carriers for the immobilization of laccase, and the laccase was immobilized on these materials by the physical adsorption method. In a typical procedure, 100 mg of SmBO_3_ support was suspended in 10 ml of phosphate buffer (pH = 7.0) containing a certain amount of laccase (about 20 mg). The mixture of the supports and laccase solution was slowly stirred at room temperature for 12 h. Subsequently, the laccase immobilized on SmBO_3_ was separated by a centrifuge. Then the samples were washed with 10 ml of buffer solution by shaking for 5 min and separated quickly using a centrifuge. The washing procedure was repeated several times until no protein was detected in the supernatant. Finally, the laccase immobilized by SmBO_3_ were stored at 4°C before using. The percentage of the immobilized laccase on the SmBO_3_ samples is in the range of 10.7% ~ 15.2%.

### Preparation of the glassy carbon electrode

Ultrasonic agitation was used to disperse 1-mg SmBO_3_-immobilized laccase into 1-ml Nafion to give a suspension (1 mg · ml^-1^). Before an experiment, the GCE was polished successively with 0.1-μm γ-Al_2_O_3_ powder, and then on a polishing cloth. Residual polishing material was removed from the electrode surface by ultrasonic agitation in concentrated HNO_3_, distilled water, and absolute ethanol. Then, the GCE was coated with 10 μl of laccase immobilized by SmBO_3_-Nafion suspension (1 mg · ml^-1^) and the solvent evaporated under room temperature for 1 h. The modified electrode was cleaned with distilled water before use.

## Results and discussion

### SEM studies

**Figure 2:**
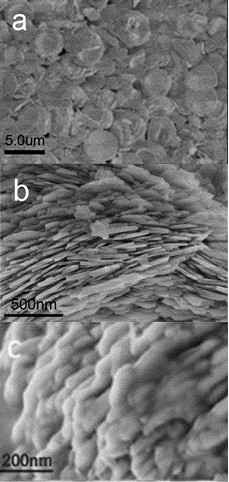
**Typical SEM images of as-prepared SmBO**_**3 **_**(a), corresponding high-magnified images (b), and immobilized laccase images (c).**

Figure 2a shows SEM micrographs of as-prepared SmBO_3_ multilayer obtained via the additive-free S-S-H method at 200°C for 36 h. Figure 2b was the corresponding high-magnified images. The multilayer shapes consist of multilayer nanosheets. These nanosheets have typical diameters of 3 ~ 5 μm while the thickness of the single layer are in the range of 10 ~ 80 nm. These microparticles are nonaggregated with narrow size distribution. The pseudo-vaterite self-assembled SmBO_3_ multilayers exhibit advantages in high-ratio surface area and analogy-graphite layer structure, which are favorable for potential application in enzyme immobilization. Figure 2c shows that the laccase was effectively filled among layers of SmBO_3_ by physical absorption. Inspired by this, we inferred the multilayer structures of SmBO_3_ suitable for immobilization of other enzymes.

### The XRD pattern analysis of as-prepared SmBO_3_ samples

**Figure 3:**
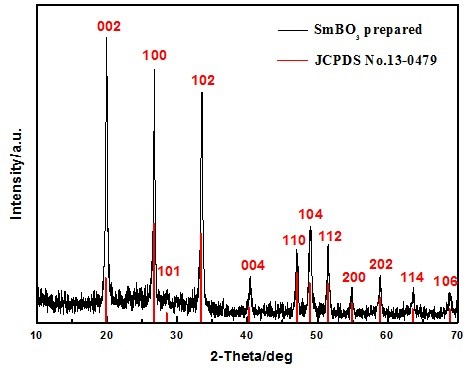
**XRD pattern of SmBO**_**3 **_**via S-S-H method at 200°C for 36 h.**

To ascertain the structure of as-prepared SmBO_3_ samples, corresponding XRD patterns of samples were investigated and shown in Figure 3. The pattern is inconsistent with aragonite-type, which are indexed in the standard pattern database listed in JCPDS. To make clear the crystal structure, the MDI Jade (5.0 Edition) software was applied to auto index the similar patterns in JCPDS. It was found that the peak positions are in accordance with the primitive-lattice hexagonal phase SmBO_3_ (No. 13-0479).

### FTIR spectra analysis

**Figure 4:**
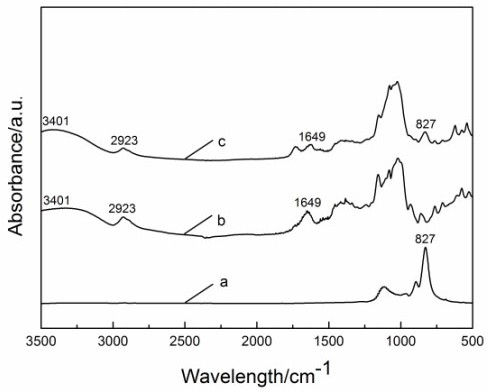
**FTIR spectra of SmBO**_**3 **_**(a), laccase (b), and SmBO**_**3**_**-immobilized lacasse (c).**

Figure 4a shows FTIR spectra of SmBO_3_ prepared via the S-S-H method at 200°C for 36 h. The absorbance peaks are assigned to the vibration mode of the ring anion B_3_O_9_^9-^. A feature of this model is that the B_3_O_9_^ 9-^ group is involving a planar ring with D3 symmetry. The assignment model is proposed in hexagonal LnBO_3_ as follows: Due to the stretching vibrations of the ring sketch of the cyclic trimeric ion and the terminal B-O and bending vibrations of them, the absorption bands in the region of 800 to 1,200 cm^-1^and below 500 cm^-1^, respectively [31-34]. To investigate the binding between the laccase and the laminated SmBO_3_ multilayers, FTIR spectra for the laminated SmBO_3_ multilayers, lacasse, and laminated SmBO_3_ multilayers with immobilized laccase were measured.

Figure 4b,c shows the FTIR spectra laccase and SmBO_3_-immobilized lacasse. Compared to the typical absorption peaks of lacasse at 3,401, 2,923, and 1,649 cm^-1^ and the main absorption peaks of SmBO_3_ at 1,110, 960, 894, and 827 cm^-1^, the absorption of SmBO_3_-immobilized lacasse include all of the above peaks. So it is evident that the laccase was successfully immobilized on SmBO_3_ nanosheets. Moreover, it can be seen from Figure 4 that the positions of lacasse and those immobilized in SmBO_3_ are nearly at the same place, suggesting that the lacasse retains its native structure in SmBO_3_-immobilized lacasse.

### Electrochemical properties

**Figure 5:**
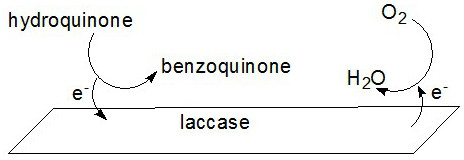
**Scheme of reactions occurring at surface of laccase-immobilized SmBO**_**3**_**-modified GCE.**

The response of laccase-immobilized SmBO_3_ nanosheets for phenolic compound detection is based on the mechanism in which a substrate (hydroquinone in this case), laccase, and oxygen are involved. The enzymatic mechanism involved in laccase-immobilized SmBO_3_ for phenolic compound detection is the same as the bare laccase [4]. Laccase as one of the multicopper oxidases contains four copper atoms and catalyzes the four-electron reduction of O_2_ to H_2_O at a trinuclear copper cluster. The catalytic process consists of the oxidation of hydroquinone by laccase followed with the reduction of O_2_ by laccase (Figure 5).

**Figure 6:**
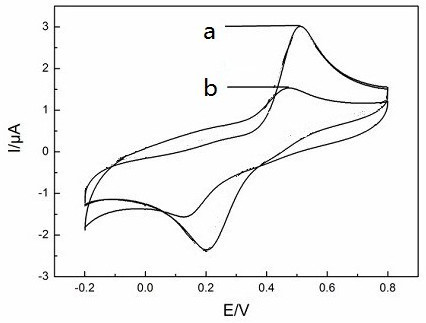
**Cyclic voltammetry of SmBO**_**3**_**-immobilized laccase (a) and bare electrode (b).** At a scan rate of 50 mV/s in pH 4.0 PBS, at room temperature with the presence of 5 × 10^-5^ mol · l^-1^ hydroquinone.

The electrochemical behaviors of laccase-immobilized SmBO_3_-modified GCE in various solutions were studied using cyclic voltammetry and the results are shown in Figure 6. The laccase-immobilized SmBO_3_-modified GCE remain its redox behaviors in pH 4.0 PBS at room temperature with the presence of 5 × 10^-5^ mol · l^-1^ hydroquinone. The anodic peak currents of laccase-immobilized SmBO_3_-modified GCE are 3.0 μA. Compared to the anodic peak current of bare electrode which is 1.48 μA, the anodic peak current of modified GCE is at least two times greater. These demonstrate that the electrode of the SmBO_3_-immobilized laccase has a better sensitivity to the substrate. At the same time, we found that the Δ*E* of laccase-immobilized SmBO_3_-modified GCE (0.51 V) is larger than bare electrode (0.47 V). According to the Gibbs-Helmholtz equation Δ*G* = -*nF*Δ*E*, Δ*G* of the laccase-immobilized SmBO_3_-modified GCE is smaller than the bare electrode. These results suggest that the reaction occurs on the laccase-immobilized SmBO_3_ electrode is much easier than the bare electrode.

### Optimal parameters

**Figure 7:**
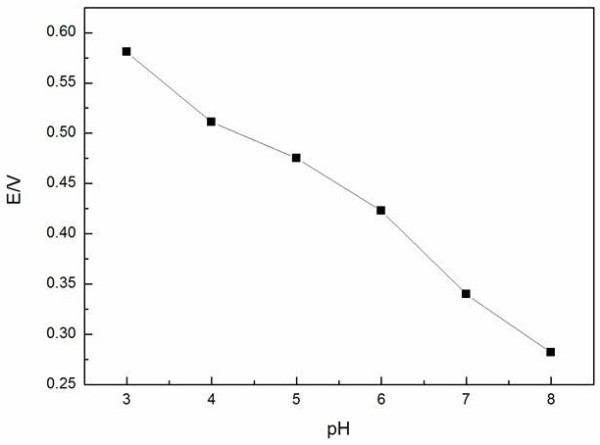
**Influence of pH on anodic peak potentials of laccase immobilized on SmBO**_**3**_**.** At a scan rate of 50 mV · s^-1^ in presence of 5 × 10^-5^ mol · l^-1^ hydroquinone, at room temperature.

**Figure 8:**
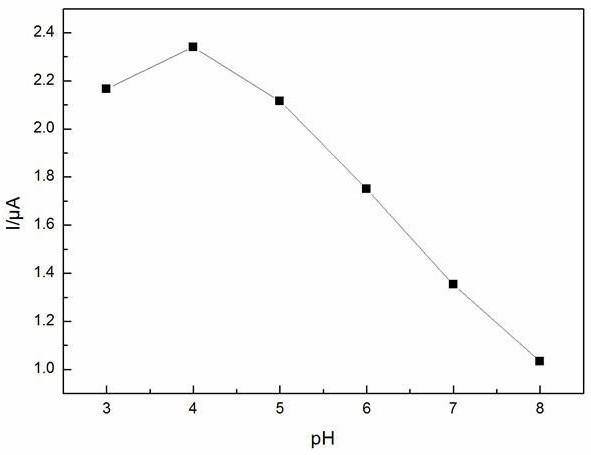
**Influence of pH on anodic peak currents of laccase immobilized on SmBO**_**3**_. At a scan rate of 50 mV · s^-1^ in presence of 5 × 10^-5^ mol · l^-1^ hydroquinone, at room temperature.

We used 0.2 mol · l^-1^ Na_2_HPO_4_ · 12H_2_O and 0.1 mol · l^-1^ C_6_H_8_O_7_ · H_2_O solutions to adjust the pH of the buffer solutions from 3.0 to 8.0. Figures 7 and 8 show the relationship between the pH values and the anodic peak potentials, the anodic peak currents from CV, respectively. The potentials shifted negatively with increasing pH value. At the same time, the anodic peak currents increased slightly with increasing pH, and when the pH exceeded 4.0, the anodic peak currents decreased immediately. It may be due to the high oxidation potentials and the serious interference at low pH values. Therefore, pH 4.0 was chosen as the optimum pH in this work.

**Figure 9:**
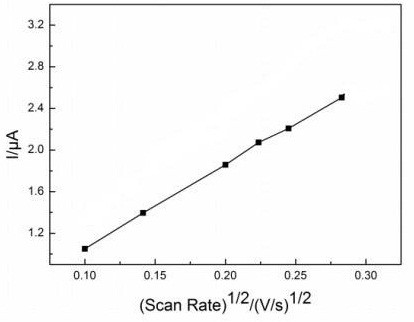
**Influence of square root of scan rate on anodic peak currents of laccase immobilized on SmBO**_**3**_. At a scan rate of 50 mV · s^-1^ in pH 4.0 PBS, at room temperature in presence of 5 × 10^-5^ mol · s^-1^ hydroquinone.

Cycle voltammograms were employed to investigate the influence of scan rate on hydroquinone oxidation at the laccase-immobilized SmBO_3_-modified electrode. The results are shown in Figure 9. At scan rates in the range of 0.01 to 0.1 V · s^-1^, the oxidative peak currents of the laccase-immobilized SmBO_3_-modified electrode in hydroquinone solution increased linearly with the square root of the scan rate, which proved that the electro-oxidation of hydroquinone was a diffusion-controlled process.

### Calibration graphs

**Figure 10:**
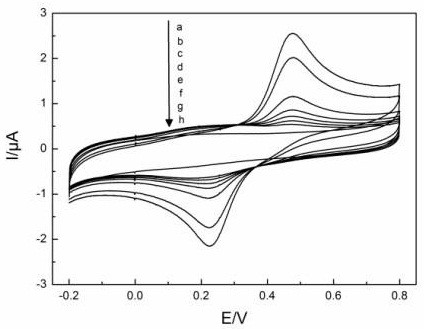
**Calibration graphs of concentration of hydroquinone of laccase-immobilized SmBO**_**3**_**-modified electrode.** a. 5, b. 3, c. 1, d. 0.8, e. 0.5, f. 0.3, g. 0.1, h. 0 × 10^-5^ mol · l^-1^.

The anodic peak currents (*I*_*p*_) of laccase-immobilized SmBO_3_-modified electrode of the CV are proportional to the concentration of hydroquinone from 1 × 10^-6^ to 5 × 10^-5^ mol · l^-1^. The picture is shown in Figure 10.

**Figure 11:**
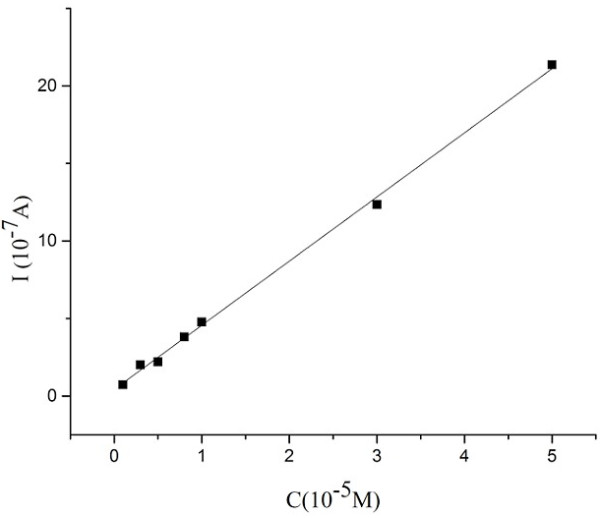
Calibration curve between catalytic current and concentration of hydroquinone in pH 4.0 PBS, at room temperature.

The calibration curve under optimal conditions is shown in Figure 11. The linear response range of laccase-immobilized SmBO_3_-modified electrode to hydroquinone concentration is from 1 to 50 μM with a correlation coefficient of 0.998 (*I* = 4.13c +0.42, *r* = 0.998). The detection limits of the compounds are estimated to be 3 × 10^-7^ mol · l^-1^.

## Conclusions

In summary, we have demonstrated a nanosensor composed of laminated samarium borate and immobilized laccase for phenol determination. These SmBO_3_ nanosheets have been successfully prepared via a mild solid-state-hydrothermal method without any surfactant or template, and laccase was successfully immobilized on these multilayers through physical adsorption method. The uniform multilayer-intersected structure could play an important role in the adsorption of laccase. This novel laccase immobilization method based on SmBO_3_ improved the performance of the laccase for phenol determination. The linear range and bioactivity of laccase-modified electrode can also satisfy the practical application. The present study has enlarged the family of support for laccase immobilization and may provide an efficient approach for phenol determination.

## Competing interests

The authors declare that they have no competing interests.

## Authors’ contributions

PH and XZ carried out the experiments and analyzed the data. PH drafted and revised the paper; QW designed and supervised the whole work. All authors read and approved the final manuscript.
